# Treatment of Child/Adolescent Obesity Using the Addiction Model: A Smartphone App Pilot Study

**DOI:** 10.1089/chi.2014.0124

**Published:** 2015-06-01

**Authors:** Robert A. Pretlow, Carol M. Stock, Stephen Allison, Leigh Roeger

**Affiliations:** ^1^ eHealth International, Inc., Seattle, WA.; ^2^ Buntain School of Nursing, Northwest University, Kirkland, WA.; ^3^ Psychiatry Department, Flinders University, Adelaide, South Australia, Australia.

## Abstract

***Background:*** The aim of this study was to test a weight loss program for young people based on an addiction treatment approach.

***Methods:*** A pilot study (*n*=43) was conducted of a 20-week child/adolescent obesity intervention based on an addiction treatment model (staged, incremental withdrawal from problem foods, snacking/grazing, and excessive amounts at meals) and implemented by a server-integrated smartphone app with health professional support. The primary outcome was standardized %overBMI measured at four time points. Secondary outcomes were participants' self-ratings of self-esteem, control over food, and the degree they turned to food when stressed. User satisfaction data were collected with an online questionnaire. Latent growth modeling techniques were used to identify independent variables and possible mediating treatment process variables associated with weight change.

***Results:*** Mean age of participants was 16 years (range, 10–21), 65% girls, and 84% Caucasian. Twenty-seven (63%) completed the program. There was a significant decrease in %overBMI over time of 7.1. There were significant improvements in participant ratings of self-esteem, control over food, and a reduction in turning to food when stressed. Males, younger participants, and participants with higher levels of program compliance achieved better weight loss. Participants who reported that calling obesity an addiction made their guilt worse experienced poorer weight loss. Females were more likely than males to report “addiction guilt,” and this partly mediated the overall gender effect.

***Conclusions:*** The staged, incremental food withdrawal approach was feasible to implement and was useful in helping reduce excessive weight, particularly among boys.

## Introduction

Obesity is an intractable health condition for millions of young people.^1^ Although modest effects for obesity treatment have been shown in younger children, this declines in older children and adolescents, and few interventions involving adolescents have produced significant long-term weight loss.^2^ New strategies are urgently needed.

In the current study, we describe a new obesity intervention for young people, which is based on an addiction treatment approach of staged, incremental withdrawal from problem foods, snacking/grazing, and excessive amounts of foods at meals. This intervention evolved from a decade of experience with a popular interactive, open-access website for overweight children/adolescents, who often described addictive patterns of eating in their bulletin board posts.^3^ These qualitative reports are complemented by emerging biological evidence that food and drugs of abuse exploit similar pathways (the dopamine and opiate systems) in the brain, ^4,5^ and that there is addictive potential of certain foods that are high in fat, sugar, and refined grains, such as potato chips and fries, sodas, and sweets.^6,7^ Further, young brains that are still developing are particularly vulnerable to addiction.^8^

The evidence that the overconsumption of some kinds of foods contributes to obesity through an addictive pathway raises the possibility of developing interventions based around techniques typically employed in traditional addiction treatments.^9,10^ An effective addiction-based obesity intervention, specifically targeted at children/adolescents, therefore has the potential to provide a treatment approach where few effective options exist.

To date, very few addiction-based obesity treatments have been developed either for adults or young people. As far as we are aware, only two other programs (“Overeaters Anonymous” and “Energy Up”) have been reported in the literature. Overeaters Anonymous is a group-based program that was created in the 1960s based on the 12-step approach of Alcoholics Anonymous. A recent survey found that approximately 70% of participants reported that they had lost weight since joining the program.^11^ The Energy Up program was developed to coach obese female high school students in the avoidance of foods containing flour, sugar, and salt, which were thought to trigger overeating.^12^ In an uncontrolled study of Energy Up, good weight loss (mean BMI change of −2.3 kg/m^2^) was achieved, although based on self-reported weights.

The key aim of the present pilot study was to examine weight loss outcomes from an addiction-based treatment method (involving staged food withdrawal) designed for child/adolescent obesity. We also hoped, through a process evaluation, to identify potential moderators of treatment effect as well as to better understand the most appealing and useful elements to children and adolescents of an addiction-based obesity intervention delivered through youth-popular smartphone technology.

## Methods

A pilot study was conducted in a convenience sample of young people who responded to newspaper and radio advertisements in Seattle, Washington, for a “Smartphone app weight loss study.”

### Procedure

Approval was obtained from the Northwest University Human Subjects Review Board. In total, 76 children and adolescents were screened for eligibility using an online application/questionnaire followed by two telephone interviews. Motivation was assessed by a self-rated 10-item scale, for example, “How much does being overweight bother you?” (response format, 1–10), summed to produce a total score (10–100) with higher scores indicating high levels of motivation. Applicants with motivation scores less than 50, or those unwilling to attend group or phone meetings or weigh their foods at meals, were excluded. Forty-three subjects were selected. Informed consent was obtained from a parent or guardian and the child/adolescent.

Each recruited participant was supplied with an iPhone 4S, a wireless Bluetooth body weight scale (Wahoo Fitness Balance Scale; Wahoo Fitness, Atlanta, GA) interfaced to the app, and a digital food scale (ONYX; American Weigh Scales, Inc., Norcross, GA). It was believed that the newer iPhone 5 had no additional features that would have boosted participant compliance and that the iPhone 4S would be less likely to be stolen. Participants were informed that they would be compensated a maximum of $200, proportional to completion of requirements of the study (daily weigh-ins, daily check-ins regarding their progress, as well as weekly phone meetings and four face-to-face group meetings).

### The Intervention Program and Smartphone App Implementation

The addiction approach intervention was implemented by an iPhone app, which was securely integrated with a network server for real-time data access and storage. The 20-week program focused on sequential withdrawal from problem foods, snacking, and excessive food amounts at meals. Participants were asked to weigh themselves daily. Weekly 15-minute phone meetings were conducted between each participant and the mentor (R.P. or C.S.), and four 2- to 4-hour face-to-face group meetings were held. Participants also were sent “eRoom” (text) messages from their mentors and were provided with the mentors' contact details (phone/e-mail). The program did not require parental involvement, but support in making the family home “problem food safe,” not buying snacks for their child, and helping with weighing food amounts was encouraged.

Problem foods were defined as specific foods for which participants felt that they had cravings, they ate when sad or stressed, they could not resist eating when immediately available, or foods they ate even when not hungry. Participants withdrew from each problem food, one by one, by total abstinence from the food for a minimum of 10 days in a row.

Participants were then asked to eliminate snacking or grazing (nonspecific foods). A snacking abstinence time interval was selected by the app—morning, afternoon, evening, or nighttime. Once the participant had successfully abstained from snacking during the respective time interval for 10 days in a row, the participant then proceeded to abstain from snacking during the next time interval, with the aim of zero snacks during the entire day.

In the final stage of the program, withdrawal from excessive food amounts at home meals was targeted. Each participant created a list of no more than 20 of his or her commonly eaten foods—including foods for breakfast, lunch, and dinner—from which typical serving amounts were weighed and recorded in the app. Once typical amounts of all frequent mealtime foods had been entered, the participant selected a mentor-guided percent to reduce amounts of all logged foods, which the app then computed. At all subsequent meals, participants accessed the current reduced amounts of each food in the app and weighed out and ate only those amounts. If weight loss had not occurred within 4 days, the app queried participants to further reduce all logged food amounts through the percent reduction feature, until weight loss ensued. If weight loss stopped, the app queried the participant to reduce the reduction percent additionally.

Peer support was included in the form of app bulletin boards and assigned “weight loss buddies” within the study group (utilizing the app's buddy chat). Buddies were matched according to age, gender, and weight.

To guard against unhealthy weight control strategies, the app's provider platform contained a range-settable alarm to notify the provider by e-mail if too much weight loss occurred for any participant in the daily weigh-ins. Possible binge eating and purging, as a reaction to restrictive eating, was monitored over the course of the intervention by means of the weekly phone meetings. If binge eating or purging were detected and restrictive eating were the cause, food restriction would be relaxed temporarily. If owing to interpersonal difficulties, the participant would be counseled on healthier means to cope with distress, such as journaling life events and feelings or calling a friend or mentor.

Further details on the components of the app are provided in Table 1.

**Table 1.** Components of the Intervention and App

Components	Why included	How operationalized in program
Progress report (weigh-in)	Self-monitoring is a key element to most behavioral weight loss programs. Technology may improve self-monitoring adherence.^13–15^	Daily weighing with Bluetooth wireless scales; app weigh-in reminders
Problem foods control panel	Addressing consumption of certain highly palatable foods is a key element in most behavioral weight loss programs.^13,14^	Staged withdrawal from each self-identified problem food by abstinence, one by one, for a period of 10 days or more, until cravings resolve
Snacking control panel	Addressing consumption of nonspecific foods eaten between meals (snacking/grazing) is a key element in most behavioral weight loss programs.^13,14^	Staged withdrawal from snacking/grazing by abstinence during progressively increasing time intervals (morning afternoon, evening, nighttime)
Food amounts control panel	Addressing consumption of excessive mealtime amounts of foods (portion control) is a key element in most behavioral weight loss programs.^13,14^	Staged withdrawal from excessive amounts by creating a list of no more than 20 frequently eaten mealtime foods from which components are weighed for breakfast, lunch, and dinner. The app gradually reduces weighed amounts in small increments, minimizing withdrawal symptoms (“brain hunger”).
My mentor area	Professional health contact can improve outcomes in obesity treatmen.^13^	Weekly scheduled telephone sessions with a health professional; frequent “eRoom” (secure text) messages
Motivation	Building motivation in weight loss programs has been shown to improve outcomes.^13^	Participants input reasons why they do not like their weight and reasons to be thinner. Information about health risks from being overweight is included in the app.
Fun activity jar	Substitute alternative behaviours for eating.^14^	In a moment of an urge to snack, participants swipe the jar and an activity idea appears to distract them from eating.
Coping skills area	Coping skill augmentation with problem solving would decrease using food as a coping mechanism.^14^	Participants list the things that worry them, then develop plans for each.
Peer support area	Online support communities can provide a mechanism for people to help one another to achieve their goals.^15^	The app provided access to the weight2rock.com bulletin boards.
Self-esteem area	Building self-esteem is an established approach to treating obesity.^13^	Participants entered their strengths, what they like about how they look, and what others like about them.
Food/emotion diary	By identifying patterns behind food and emotions, problems with emotional eating can be addressed.^14^	Participants entered foods eaten at each meal and snacks and indicated how sad versus happy they felt at each meal/snack.
My buddy area	The provision of social support by peers can facilitate behavioral change.^13,15^	Participants were matched with “buddies” to chat with during the study.
Vicious cycles area	Overeating may be secondary to depressed mood, and weight gain produces more negative affect.^13^	Participants select vicious cycles to which they relate and type plans to break the cycle at any point.

### Data Collection Procedures

Demographic information was collected from the young people at the time of informed consent. Weight and height data were collected by trained senior nursing students during scheduled face-to-face meetings at Northwest University (Kirkland, WA). These occurred at baseline (0 days), 40 days, 90 days and 140 days (program completion). Height was measured using a Health-O-Meter stadiometer (Continental Scale Corp., Bridgeview, IL). Weight was measured on a self-calibrating, 500-pound capacity Fairbanks digital scale. A second Fairbanks scale was used to validate accuracy of the first Fairbanks scale during the weighing process. Participants wore minimal everyday clothing (but not shoes) during the height and weight measurements. Participants also completed an online self-report questionnaire at the beginning and completion of the program.

### Primary and Secondary Outcomes

The primary outcome was %over BMI. Weight and height were used to calculate BMI (kg/m^2^). Percent overBMI was calculated using the lambda-mu-sigma method^16^ based on the CDC 2000 growth curves.^17^ Percent overBMI provides a value relative to the 50th percentile BMI for the appropriate age and gender, where positive values are over the 50th percentile and negative values are under the 50th percentile. It is calculated as BMI – BMI at 50th percentile for age and gender/BMI at 50th percentile×100. Percent overBMI is readily interpretable,^18^ is sensitive to changes in BMI throughout the full range of overweight values, and is favored in studies that seek to examine predictors for change that include baseline values as a predictor.^19^

Secondary outcomes were conceptualized as proximal outcomes that were hypothesized to link the program to the primary outcome of longer-term weight loss (see Table 2). Specifically, we hypothesized that better control over food, a reduction in turning to food when stressed, and improved levels of self-esteem would be associated with better levels of longer-term weight loss.

**Table 2.** Program Implementation, Satisfaction, and Outcome Variables

Variables	Description	Coded
Program implementation and satisfaction		
Problem foods	Identify problem foods.	Number of problem foods identified and withdrawn from
Snacking	Participant report of snacking	Frequency of snacks per day at start of study compared to end
Amounts of foods at home meals	Reduction percent of home meal foods at start of study compared to end	Amounts (weights) of home meal foods consumed at start vs. end
Addiction guilt	Does calling overweight/obesity an addiction affect your guilt or self-blame about your weight? (response options: “Makes it worse”; “Makes it better”; “No difference”)	0=makes it better/no difference; 1=makes it worse
Effort	Participants' rating of how much effort they put into the study?	1=not much to 5=a lot
Satisfaction with app	For each component (and overall), participants were asked to rate how helpful the app was for losing weight.	1=not much to 5=a lot
Satisfaction with mentor	Participant rating of the helpfulness of mentors (R.P. and C.S.)	1=not much to 5=a lot
Mentor rating of compliance	Mentor rating of participant compliance with program	1=very poor to 5=very good
Outcome variables		
Primary		
%overBMI	A value relative to the 50th percentile standardized BMI. Positive values are over the 50th percentile and negative values are under the 50th percentile.	BMI – BMI at 50th% for age and gender/BMI at 50th percentile×100
Secondary		
Control	Before you started the study, how much were you able to control your eating? How much are you able to control your eating now, at the end? (rated 1=not much to 5=a lot)	Difference between before and after ratings
Self-esteem	Rate your self-esteem at the beginning of this study? Rate your self-esteem now, at the end of this study? (rated 1=really bad to 5=really good)	Difference between before and after ratings
Stress	How much were you turning to food when upset or stressed at beginning of study? How much are you turning to food when upset or stressed now? (rated 1=not at all to 5=the most)	Difference between before and after ratings

### Program Performance and Satisfaction

Data relating to participant performance through the app was tracked remotely and stored on central servers. Data are reported (see Table 2) on the three key components specific to the underlying addiction model implemented in the intervention: (1) whether participants could identify “problem foods” and withdraw from them; (2) whether participants were able to eliminate snacking; and (3) the extent to which participants were able to reduce the amounts of foods consumed at home meals.

Participants also rated the usefulness of the different components of the program for losing weight, the helpfulness of the mentors (C.S. and R.P.), the amount of effort they put into the study, and whether the addiction model approach used in the program had caused their guilt about being overweight to increase. Investigators removed participants from the study owing to noncompliance with program key requirements, specifically repeated absenteeism from phone meetings or group meetings, repeated failure to respond to voicemail or eRoom messages from mentors, or refusal to weigh their foods at meals. Mentors rated participant compliance at the conclusion of the study.

### Statistical Analysis

Logistic regression was used to identify possible predictors of program completion from a range of independent variables, including gender, age, race, family type, socioeconomic status (SES), and motivation. Changes in the secondary outcomes were assessed using paired-sample *t*-tests.

Latent growth curve analysis (LGCA) was used to assess the primary outcome of weight loss. The LGCAs were performed using %overBMI calculated at each of the four time points (0, 40, 90, and 120 days) as the observed dependent variables for the 27 participants who completed the program. The analyses presented therefore are per protocol (subjects who completed the program), as opposed to intent to treat. In the LGCA models, the latent intercept variable was centered relative to scores at the first time point (0 days) representing the initial status of the growth curve. The linear slope reflects the growth trajectory across the time points.

Four LGCAs were performed: (1) A null model (no predictors) was estimated to examine the fit of the linear model and to gauge the extent of interindividual differences in initial weight status and weight change over time; (2) a series of LGCA univariate conditional models were performed to identify possible predictors of weight change; and (3) a multivariate model was estimated. Using this multivariate model, a final (4) LGCA analysis was performed to examine whether secondary outcomes or program implementation variables mediated the relationship between independent variables and weight change.^20^ Model fit was evaluated drawing from structural equation modeling recommendations specifically developed for LGCA.^21^

Descriptive statistics and logistic regression analyses were conducted using Stata software (version 12; StataCorp LP, College Station, TX). LGCAs were performed in MPlus 7.2 using the wide format approach with the maximum likelihood estimator.^22^

## Results

A total of 43 children/adolescents entered the study with a mean age of 16.0 years. The majority of participants were girls (65%) and of white ethnicity (83.7%). Approximately two thirds (62.8%) were overweight/obese (85th–98th BMI percentile) and one third (37.2%) were severely obese (>98th BMI percentile; see Table 3).

**Table 3.** Baseline Characteristics

Characteristics	Male (*N*=15) *N* (%)	Female (*N*=28) *N* (%)	Total (*N*=43)
Age			
10–14	7 (46.7)	11 (39.3)	18 (41.9)
15–17	5 (33.3)	10 (35.7)	15 (34.9)
18–21	3 (20.0)	7 (25.0)	10 (23.3)
Mean (SD)	15.7 (0.76)	16.1 (0.53)	16.0 (0.43)
Race			
Caucasian	13 (86.6)	23 (82.1)	36 (83.7)
Black	2 (13.3)	2 (7.1)	4 (9.3)
Latino	0 (0.0)	2 (7.1)	2 (4.7)
Asian	0 (0.0)	1 (3.6)	1 (2.3)
Family type			
Living with both parents	9 (60.0)	12 (43.0)	21 (49.0)
Single or step family	6 (40.0)	16 (57.0)	22 (51.0)
Socioeconomic status			
Low	6 (40.0)	13 (46.0)	19 (44.0)
Middle/high	9 (60.0)	15 (54.0)	24 (56.0)
Absent from school, days			
0–2	8 (53.3)	16 (57.1)	24 (55.8)
>2	7 (46.7)	12 (42.9)	19 (44.2)
Mean (SD)	2.9 (0.93)	3.6 (0.68)	3.9 (0.53)
Motivation			
Low	6 (40.0)	7 (25.0)	13 (30.2)
Medium	5 (33.3)	10 (35.7)	15 (34.9)
High	4 (26.7)	11 (39.3)	15 (34.9)
Mean (SD)	87.8 (2.2)	88.3 (1.5)	88.1 (1.2)
BMI (percentile)			
Overweight/obese (85th–98th)	5 (33.3)	22 (78.6)	27 (62.8)
Severe obesity (>98th)	10 (66.7)	6 (21.4)	16 (37.2)
Mean BMI percentile (SD)	0.99 (0.00)	0.98 (0.00)	0.98 (0.00)
Percent over BMI (mean, SD)	90.1 (10.8)	70.5 (3.7)	77.4 (4.6)

SD, standard deviation.

### Program Attrition

Of 43 participants entering the study, 27 (62.7%) completed. The 16 participants who did not complete were either removed by investigators for noncompliance (8 participants), voluntarily withdrew (7 participants), or moved (1 participant). In logistic regression analyses, no statistically significant predictors of attrition were found across a range of independent variables, including gender, age, race, family type, SES, or baseline motivation.

### Problem Foods, Snacking, and Meal Amounts Withdrawal

The majority (25 of 27; 89%) of participants who completed the program were able to identify one or more specific problem foods. Of the 25 subjects who could identify one or more problem foods, only 1 participant was unable to successfully withdraw (cravings resolved) from at least one problem food. Two participants claimed to have no specific problem foods for which they had cravings or particular difficulty resisting. Thus, they immediately proceeded to withdrawal from snacking (nonspecific foods), followed by withdrawal from excessive amounts at home meals.

The majority (70%) of participants completely eliminated snacking, whereas 30% reduced (but did not completely eliminate) the frequency of snacking. This was accomplished by distractions (*e*.*g*., fun activities), avoidance of triggers (*e*.*g*., prevention of boredom or avoiding the kitchen), and keeping their hands busy while watching television.

Nearly all (26 of 27: 96%) participants reduced the weighed amounts of foods consumed at home meals, on average, to 51.1% of starting amounts. When their amounts reached a level of roughly 50–60% of starting amounts, approximately one fourth (27%) of participants admitted to sometimes consuming additional food once they had consumed their weighed amounts at a meal.

With a few notable exceptions, withdrawal from problem foods and snacking was associated with minimal withdrawal symptoms. However, withdrawal from excessive food amounts at meals was associated with significant withdrawal symptoms consisting of nagging urges, agitation, even anger, but rarely physical hunger (stomach growling, emptiness). Nevertheless, participants interpreted food withdrawal symptoms as “hunger.”

### Program Satisfaction

Average satisfaction rating of the program overall was 3.11 (1=not very helpful to 5=very helpful) with the components relating to daily weighing, problem food control, food amounts, snacking control, and mentor support receiving the highest endorsement (see Table 4). Approximately half (48.1%) of participants indicated that calling obesity an addiction made their guilt about their weight worse. There was a substantial gender difference (χ^2^(3)=7.42; *p*<0.01) with respect to “addiction guilt,” with 66.7% (12 of 18) of females indicating that their guilt was worse compared to only 11.1% (1 of 9) of male participants.

**Table 4.** Program Implementation and Satisfaction

	Male (*N*=9) Mean (SE)	Female (*N*=18) Mean (SE)	Total (*N*=27) Mean (SE)
Helpfulness of app areas^a^			
Progress report (weigh-in)	3.11 (0.48)	3.89 (0.25)	3.63 (0.24)
Problem food panel	3.38 (0.56)	3.67 (0.27)	3.58 (0.25)
Food amount panel	3.67 (0.41)	3.33 (0.23)	3.44 (0.20)
Snacking control panel	3.00 (0.47)	3.38 (0.33)	3.24 (0.27)
My mentor area	2.56 (0.47)	3.39 (0.27)	3.11 (0.27)
Fun activity jar	2.56 (0.47)	2.89 (0.38)	2.78 (0.29)
Coping skills area	1.89 (0.35)	2.83 (0.24)	2.52 (0.21)
Peer support area	1.89 (0.39)	2.83 (0.31)	2.52 (0.25)
Self-esteem area	1.89 (0.31)	2.67 (0.34)	2.41 (0.26)
Food/emotion diary	1.78 (0.43)	2.50 (0.32)	2.26 (0.26)
My buddy area	1.11 (0.11)	2.50 (0.34)	2.04 (0.26)
Vicious cycles area	1.89 (0.26)	1.94 (0.29)	1.93 (0.21)
App overall	3.00 (0.24)	3.17 (0.20)	3.11 (0.15)
Satisfaction with mentor (C.S.)^a^	3.11 (0.31)	3.94 (0.21)	3.67 (0.18)
Satisfaction with mentor (R.P.)^a^	2.89 (0.35)	3.39 (0.24)	3.22 (0.20)
Participant rating of their effort^a^	2.78 (0.32)	3.61 (0.22)	3.33 (0.19)
Mentor rating of compliance^a^	3.56 (0.41)	2.94 (0.24)	3.15 (0.21)

	*N* (%)	*N* (%)	*N* (%)
Does calling obesity an addiction affect your guilt?			
Makes it worse	1 (11.1)	12 (66.7)	13 (48.2)
No difference	7 (77.8)	5 (27.8)	12 (44.4)
Makes its better	1 (11.1)	1 (5.6)	2 (7.4)

^a^ Rated on a 1- to 5-point scale.

SE, standard error.

### Primary and Secondary Outcomes

**Figure 2.**
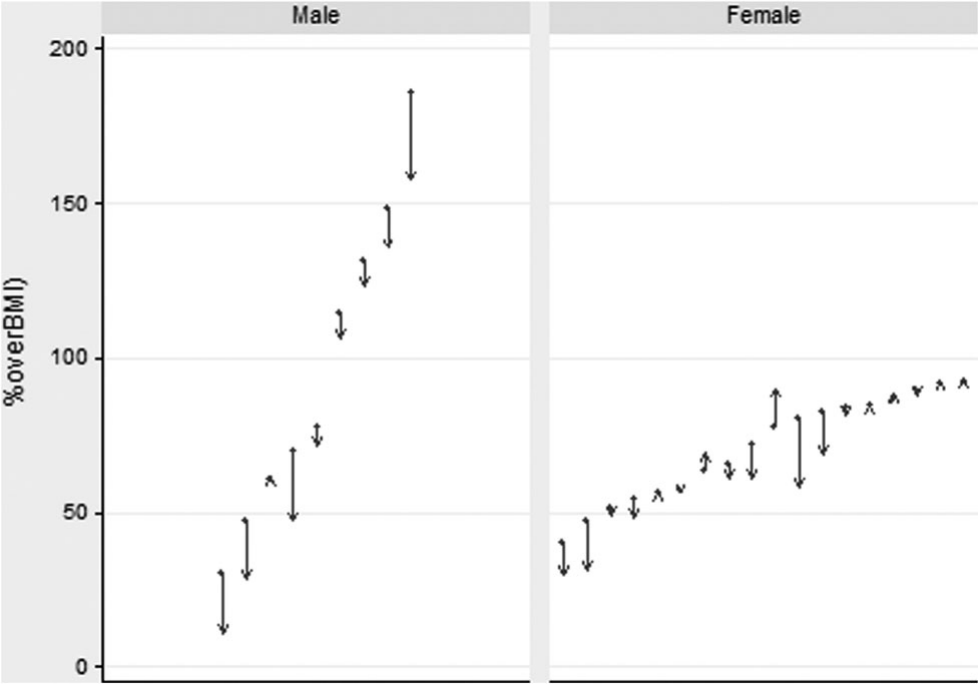
**Weight change %overBMI.**

Average weight in kilograms (kg) of the 27 participants who completed the program at baseline and at program completion was: males (113.7:108.7 kg) and females (92.1:91.3 kg). Expressed as %overBMI: males (95.9:82.6 %overBMI) and females (70.9:67.1 %overBMI). Figure 2 plots initial and program completion %overBMI for each of the 27 subjects. The figure highlights the considerable variability between participants, both with respect to their initial weights and weight change.

A null LGCA model (no predictors) was estimated to examine whether the pattern of %overBMI change was adequately represented as a linear function (Table 5). Fit indices indicated a very good fit for this linear model (χ^2^(6)=4.72; *p*=0.58; root mean square error of approximation [RMSEA]<0.01; comparative fit index [CFI]=1.0; Tucker-Lewis index [TLI]=1.0). In the null model, the average score of %overBMI at intercept (baseline) was significant (unstandardized estimate=79.02; *p*<0.01). There was a statistically significant (unstandardized estimate=–0.051; *p*<0.01) decrease in %overBMI from baseline to program completion of 7.14 (140 days at 0.051 per day).

**Table 5.** LGCAs of %overBMI

	Estimate	SE	Est/SE	*P* value
Null model^a^				
Intercept	79.02	6.27	12.60	<0.01
Slope	−0.051	0.013	−3.94	<0.01
Residual var–intercept	1061.46	289.14	3.67	<0.01
Residual var–slope	0.004	0.001	2.77	<0.01
Cov–intercept and slope	−0.35	0.43	−0.82	0.41
Predictors of weight change^b^				
Gender–intercept	−0.39	0.16	−2.36	0.02
Gender–slope	0.71	0.13	5.32	<0.01
Age–slope	0.42	0.20	2.08	0.04
Race–slope	0.10	0.23	0.42	0.68
Family type–slope	0.08	0.21	0.36	0.72
Socioeconomic status–slope	−0.10	0.19	−0.51	0.61
Absent from school–slope	0.25	0.21	1.19	0.23
Motivation at baseline–slope	0.22	0.24	0.90	0.37
Control overeating–slope	−0.15	0.21	−0.68	0.50
Self-esteem–slope	−0.31	0.20	−1.56	0.12
Stress–slope	−0.20	0.21	−0.95	0.34
Helpfulness of mentor (B.P.)–slope	0.20	0.22	0.92	0.36
Helpfulness of mentor (C.S.)–slope	−0.02	0.22	−0.07	0.94
Effort–slope	0.33	0.20	1.64	0.10
Addiction guilt–slope	0.59	0.15	4.07	<0.01
Compliance with program–slope	−0.68	0.12	−5.71	<0.01
Final model^b^				
Gender–intercept	−0.39	0.16	−2.36	0.02
Gender–slope	0.42	0.17	2.41	0.02
Age–slope	0.29	0.17	1.75	0.08
Addiction guilt–slope	0.35	0.16	2.19	0.03
Compliance with program–slope	−0.51	0.17	−2.95	<0.01
Addiction on gender	0.52	0.14	3.76	<0.01
Mediation test^b^				
Total	0.70	0.13	5.59	<0.01
Indirect	0.21	0.10	2.16	0.03
Direct (slope to gender)	0.48	0.16	2.96	<0.01

^a^ Unstandardized estimates.

^b^ Standardized estimates.

LGCA, latent growth curve analysis; SE, standard error; Est, estimated; var, variance; Cov, covariance.

The residual variances of the intercept (unstandardized estimate=1061.46; *p*<0.01) and slope (unstandardized estimate=0.004; *p*<0.01) for %overBMI showed statistically significant interindividual differences, both in initial weight status and in change over time. The negative residual covariance between intercept and slope for %overBMI suggests that higher %overBMI at baseline was associated with higher levels of weight loss, but this was not statistically significant (unstandardized estimate=−0.35; *p*=0.41).

Participants reported positive improvements across secondary outcomes. Participants reported that they were better able to control their eating (*t*(26)=13.45; *p*<0.01) after the program (mean=4.07; standard error [SE]=0.09), compared to at the beginning of the program (mean=2.11; SE=0.14). Self-esteem improved (*t*(26)=3.41; *p*<0.01) from baseline (mean=2.78; SE=0.19) to program completion (mean=3.59; SE=0.17). Participants were less likely to turn to food when stressed (*t*(26)=−6.53; *p*<0.01) from baseline (mean=1.93; SE=0.18), compared to program completion (mean=3.22; SE=0.22).

### Predictors of Weight Change

A series of univariate conditional LGCA models were estimated to test for an interaction effect between weight at initial status and trajectory on independent variables, secondary outcomes, and treatment implementation variables. The results from these models are shown in Table 5 (predictors of weight change panel). The results indicated a statistically significant gender difference (standardized estimate=−0.39; *p*=0.02) at initial status, with males recording higher levels of %overBMI at baseline, compared with females. Males (standardized estimate=0.71; *p*<0.01) and younger (standardized estimate=0.42; *p*=0.04) participants achieved better levels of weight change, compared to females and older participants.

Participants who reported that the addiction model did not affect their guilt about their weight (standardized estimate=0.59; *p*<0.01) and those rated with high program compliance by their mentor (standardized estimate=0.68; *p*<0.01) achieved better levels of weight loss, compared to participants who reported addiction guilt or were rated with poorer levels of compliance.

**Figure 1.**
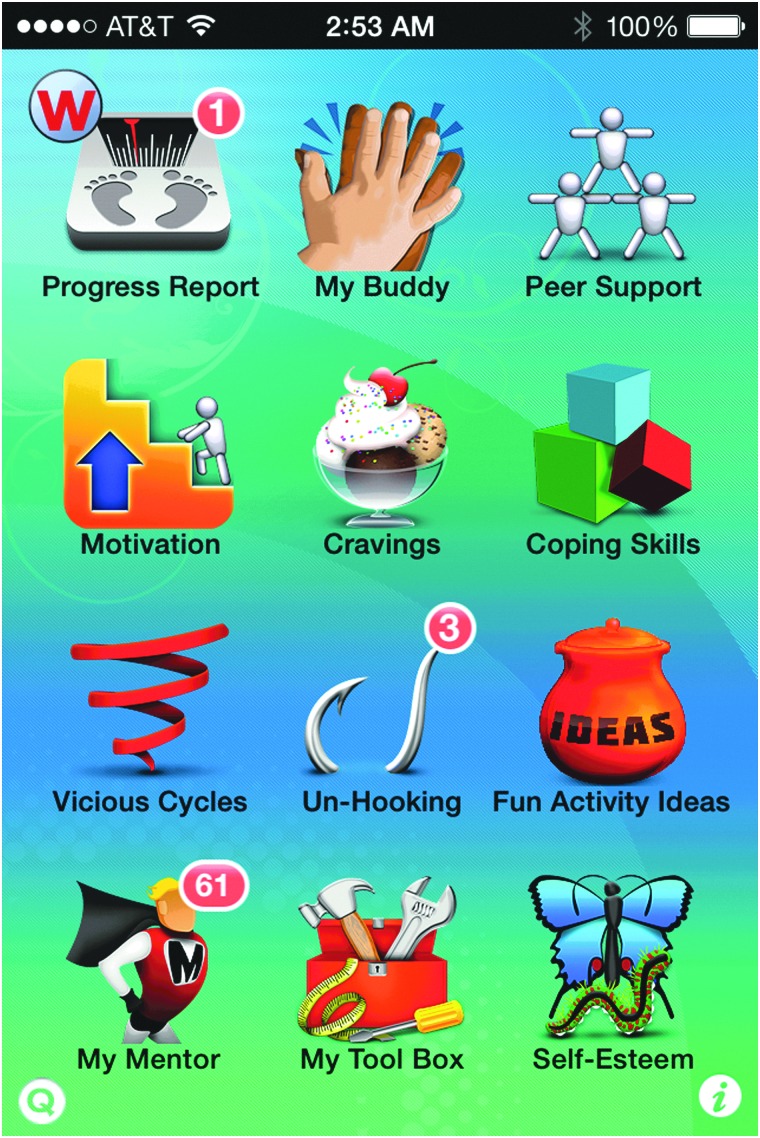
**Screen shot of front app page.**

### Final Model of Weight Change and Mediation Test

Results from the univariate analyses indicated that gender, age, addiction guilt, and program compliance were associated with weight change. A model with these variables entered simultaneously was estimated (Table 5, final model). This model provided an acceptable fit to the data: (χ^2^(22)=35.7; *p*=0.03; RMSEA=0.15; CFI=0.96; TLI=0.96).

A mediation analysis was performed to test whether the effect of the independent variables (gender and age) was mediated through the program implementation variables of addiction guilt and program compliance. Bivariate analyses indicated nonsignificant associations between age and the two program implementation variables (addiction guilt/program compliance) as well as a nonsignificant association between gender and program compliance. Consequently, these three possible mediation effects were not pursued. There was, however, a significant relationship between gender and addiction guilt. Sixty-seven percent of females, but only 11% of males, reported that calling obesity an addiction made their guilt worse.

**Figure 3.**
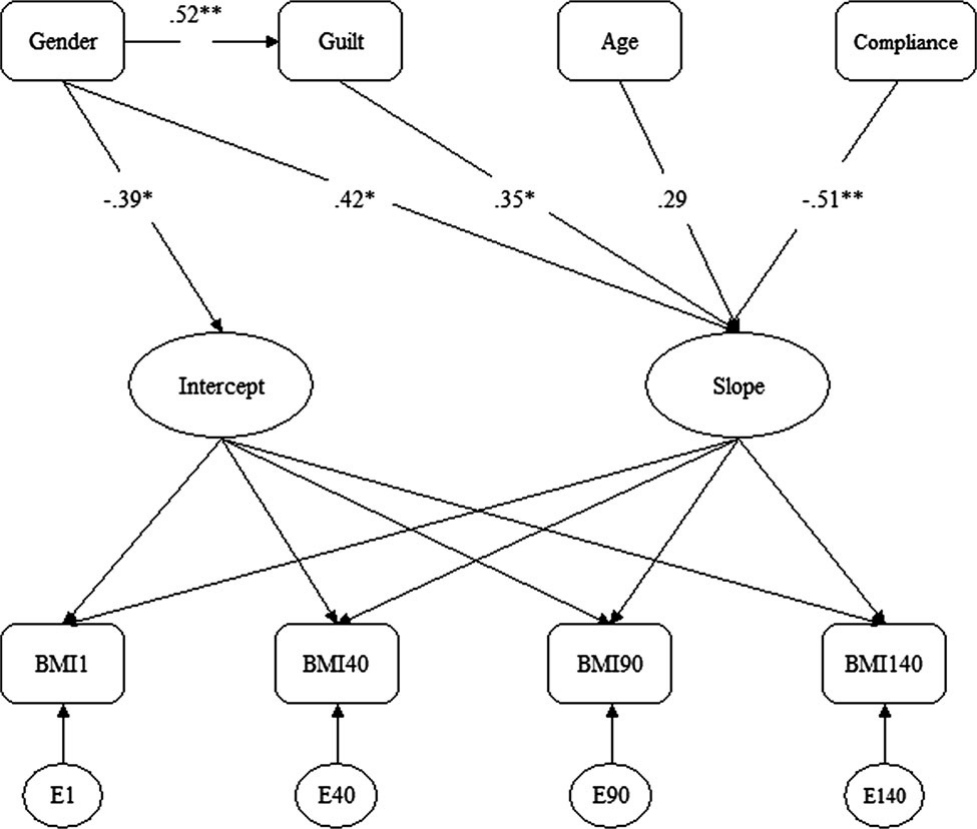
Latent growth curve analysis path diagram for mediation model. *p<0.05; **p<0.01.

The possibility that addiction guilt (a treatment implementation variable) mediated the gender effect was formally tested in a separate analysis, and the indirect effect of gender through addiction guilt was found to be significant (standardized estimate=0.21; *p*=0.03). The ratio of the indirect effect to the total effect (0.21/0.70) indicates that nearly one third (0.30) of the total effect of gender (independent variable to weight change trajectory) is through the mediating variable (addiction guilt), and approximately two thirds of the total effect is direct (gender to weight change trajectory). Figure 3 presents an LGCA path diagram for the final mediation model.

## Discussion

The present study examined weight loss outcomes and conducted a process evaluation on an addiction model-based obesity intervention that utilized staged, incremental withdrawal from problem foods, snacking, and excessive portion sizes at home meals. We found that the majority of participants were able to identify specific problem foods, eliminate urges by withdrawal from these specific problem foods, eliminate or at least reduce the frequency of snacking (nonspecific foods), and reduce the weighed amounts of foods consumed at meals. Withdrawal from problem foods and snacking were not associated with significant withdrawal symptoms. However, significant withdrawal symptoms were observed with food amounts withdrawal, which mimicked symptoms described anecdotally in case studies.^23^

Consuming more than their weighed amounts at meals by some participants, once their food amounts had reached a substantial reduction level, may represent self-compensation for food withdrawal symptoms. Detecting and managing such withdrawal symptoms (*e*.*g*., using distractions) and convincing participants that the withdrawal symptoms would soon abate may help prevent this undermining of the food-weighing process.

Incorporating relapse prevention/reinforcing the importance of sustained abstinence from snacking in the intervention would have potential benefits as well. Zero snacking greatly simplifies reducing amounts at meals by avoiding the “seesaw” effect of snacking as self-compensation when reducing food amounts. Future research is required to examine whether it is possible to identify individuals for whom the zero-snacking approach is most efficacious or for whom it is not suitable.

On the basis of our results, we believe that the approach of staged, incremental withdrawal from hyperpalatable foods, nonspecific snacking, and excessive amounts at meals is feasible to undertake when supported by a health professional. The elements of the program reported by participants as most useful for losing weight were self-monitoring of weight, food portion and snacking control, and, finally, mentor (health professional) support. Contrary to our expectations, participant engagement was minimal in the app's areas of peer support (buddy chat and bulletin boards), building self-esteem, keeping a food-emotion diary, and addressing vicious cycles. Participants reported that they were not comfortable interacting with another participant whom they did not know, particularly in regard to their weight. Planned interactions between participants at group meetings failed to improve app peer support utilization.

Overall, approximately two thirds of participants completed the 20-week program. This is consistent with a figure commonly reported in the literature for attrition from pediatric weight loss programs.^24^ On average, weight loss was in the order of 7.1 %overBMI from baseline to program completion. There were considerable interindividual differences between participants at baseline and in terms of weight loss. Younger participants and males achieved better weight loss than older participants and females.

Our age-related finding is consistent with the literature that the effectiveness of pediatric obesity treatment is modest in younger children and declines in older children and adolescents.^2^ In the adolescent weight loss literature, few gender differences in outcomes have been reported with regard to food interventions.^2^

The addiction model intervention works best when motivation is high, given that some degree of withdrawal symptoms must be tolerated, at least briefly. As in nearly all weight loss programs, motivating participants was challenging, even after participants had been selected on the basis of self-reported high levels of motivation. For the adolescents, the use of technology (iPhone and digital scales) along with financial compensation may have contributed, at least initially, to their willingness to take part in the study. In this respect, the context for the intervention will differ from typical addiction or obesity treatment provided in routine clinical settings, where expensive technologies and financial assistance are unable to be provided. It should also be acknowledged that participants returned their iPhones and digital scales on program completion and, as a result, motivation to continue using the techniques learned in the program may wane. Further research is needed to determine the best methods for inspiring youth to engage in the food withdrawal process. Our findings, that at the completion of the program participants felt that they had better control over food, a reduction in turning to food when stressed, and improved levels of self-esteem, are encouraging. Longer follow-up, however, is required to examine whether this translates to sustained weight loss.

Constructs such as “moderation” and “healthy eating” were felt to be too vague as both short- and long-term goals for this group, who had great difficulty with temptation (*e*.*g*., deciding what and how much to take). Rather, a quantitative approach of adhering to specific amounts and zero snacking is more achievable.

The results from the mediation analysis indicated that participants who reported that the process of identifying addictive eating made their guilt worse (addiction guilt) experienced poorer outcomes. It is striking that most girls (66.7%) indicated that their guilt worsened, compared to only 11.1% of boys. These gender differences in addiction guilt partly explained the gender differences in weight loss (with girls having poorer weight loss outcomes). We believe this finding is important and that future addiction-based programs should take into account the possibility of increasing guilt among females. Subsequent versions of this app will monitor addiction guilt and refer affected users to a new treatment module.

There are several limitations to the current study. The sample size was relatively small and causal inferences are not possible because it was an uncontrolled study. Formal cost-effectiveness data were not collected and further intervention development is required to facilitate broader dissemination.

## Conclusions

This study provides preliminary evidence for the feasibility and acceptability of staged food withdrawal as an addiction-based treatment model for obesity among young people, which can be delivered by health professionals through a youth-popular smartphone platform. Adapting the program for face-to-face delivery offers a forthcoming direction for the intervention. This approach has considerable potential to address a critical treatment gap in childhood obesity, especially for boys. Future programs need to investigate tailored techniques to the addiction approach for girls and older adolescents.
